# VCP/p97 Is a Proviral Host Factor for Replication of Chikungunya Virus and Other Alphaviruses

**DOI:** 10.3389/fmicb.2019.02236

**Published:** 2019-09-24

**Authors:** Guillaume Carissimo, Yi-Hao Chan, Age Utt, Tze-Kwang Chua, Farhana Abu Bakar, Andres Merits, Lisa F. P. Ng

**Affiliations:** ^1^Singapore Immunology Network, Agency for Science, Technology and Research (A^∗^STAR), Singapore, Singapore; ^2^NUS Graduate School for Integrative Sciences and Engineering, National University of Singapore, Singapore, Singapore; ^3^Institute of Technology, Faculty of Science and Technology, University of Tartu, Tartu, Estonia; ^4^School of Biological Sciences, College of Science, Nanyang Technological University, Singapore, Singapore; ^5^Department of Biochemistry, Yong Loo Lin School of Medicine, National University of Singapore, Singapore, Singapore; ^6^Institute of Infection and Global Health, University of Liverpool, Liverpool, United Kingdom

**Keywords:** VCP, VCP/p97, alphavirus, chikungunya, host factor, proviral factor

## Abstract

The evolutionarily conserved AAA+ ATPase valosin-containing protein (VCP) was previously shown to be a proviral host factor for several viruses from different viral families such as *Flaviviridae, Picornaviridae*, and *Herpesviridae*. VCP was shown to affect trafficking of Sindbis virus receptor and functions as a component of Semliki Forest virus (SFV) replicase compartment. However, the role of this cellular protein was not evaluated during replication of alphaviruses including chikungunya virus (CHIKV). Using siRNA, chemical inhibitors, and trans-replication assays, we show here that VCP is a proviral factor involved in the replication of CHIKV. Immunofluorescence assays confirmed that VCP co-localized with non-structural replicase proteins but not with dsRNA foci possibly due to VCP epitope unavailability. VCP pro-viral role is also observed with other alphaviruses such as o’nyong’nyong virus (ONNV) and SFV in different human cell lines. VCP proviral roles on several viral families now extend to replication of alphaviruses CHIKV and ONNV, emphasizing the pivotal role of VCP in virus–host interaction biology.

## Introduction

The valosin-containing protein (VCP), also named p97, is a member of the hexameric AAA+ ATPase family and is highly conserved across all domains of life (Erzberger and Berger, 2006; Barthelme and Sauer, 2016). The molecular function of VCP is ATP-driven protein unfolding (Ye et al., 2017; van den Boom and Meyer, 2018). Depending on the different associated co-factors (Hanzelmann and Schindelin, 2017), it is involved in many cellular pathways, ranging from endoplasmic reticulum (ER) and mitochondrial associated protein degradation (Taylor and Rutter, 2011; Wolf and Stolz, 2012), ubiquitin proteasome system (UPS) (Meyer et al., 2012), chromatin structure regulation (Torrecilla et al., 2017), DNA break repair (Torrecilla et al., 2017), DNA replication (Ramadan et al., 2017), NF-κB activation (Schweitzer et al., 2016), stalled ribosome turnover (Verma et al., 2013), endomembrane fusion (Zhang and Wang, 2015), full capacity of autophagy and lysosomal system (Papadopoulos and Meyer, 2017), and lipid droplet turnover (Olzmann et al., 2013).

Valosin-containing protein has been reported to be a pro- or anti-viral host factor for several viruses, such as picornaviruses (Arita et al., 2012; Wu et al., 2016; Wang et al., 2017), flaviviruses (Yi et al., 2016; Phongphaew et al., 2017; Yi and Yuan, 2017), coronaviruses (Wong et al., 2015), an herpesvirus (Lin et al., 2017), a phlebovirus (Brahms et al., 2017), and even an insect nucleopolyhedrovirus (Lyupina et al., 2018). In the case of picornaviruses (single-stranded RNA positive, ssRNA+), VCP has been implicated as a pro-viral factor in the replication of poliovirus and EV71 and several viral proteins showed direct interaction (Arita et al., 2012; Wang et al., 2017), while knock-down of VCP enhanced Aichi virus (Arita et al., 2012). VCP have been described as an important host factor for several flaviviruses (ssRNA+) West Nile virus replication (Phongphaew et al., 2017), as well as a component of Hepatitis C virus (HCV) and shown to prevent HCV replicase aggregation (Yi et al., 2016; Yi and Yuan, 2017).

In the specific case of ssRNA+ alphaviruses (family *Togaviridae*), VCP was shown to be a proviral factor for Sindbis virus (SINV) by trafficking SINV receptor NRAMP2 to the plasma membrane (Rose et al., 2011; Panda et al., 2013). However, for chikungunya virus (CHIKV) and other alphaviruses, binding and entry is NRAMP2 independent (Rose et al., 2011). In addition, VCP was later identified by mass spectrometry as a replication complex-associated protein during Semliki Forest virus (SFV) infection (Varjak et al., 2013). However, the role of this AAA+ ATPase was not further investigated during the replication of alphaviruses.

In this study, using a combination of knockdowns and chemical inhibitors with time-of-addition assays, VCP is shown to be an important proviral factor necessary for replication of several isolates of CHIKV, O’nyong’nyong virus (ONNV), and SFV. Using a CHIKV trans-replication system, VCP was verified to be an important factor required during RNA replication of the virus life cycle, but not for the stability of the non-structural proteins (nsPs). We also showed that VCP co-localized with several nsPs. Elucidating the molecular mechanisms involved in VCP proviral role, including its viral interacting protein partners, will be crucial in developing therapies targeting VCP pro-viral functions potentially efficient on several viral families.

## Results

### VCP Knockdown Inhibits CHIKV Replication *in vitro*

**FIGURE 1:**
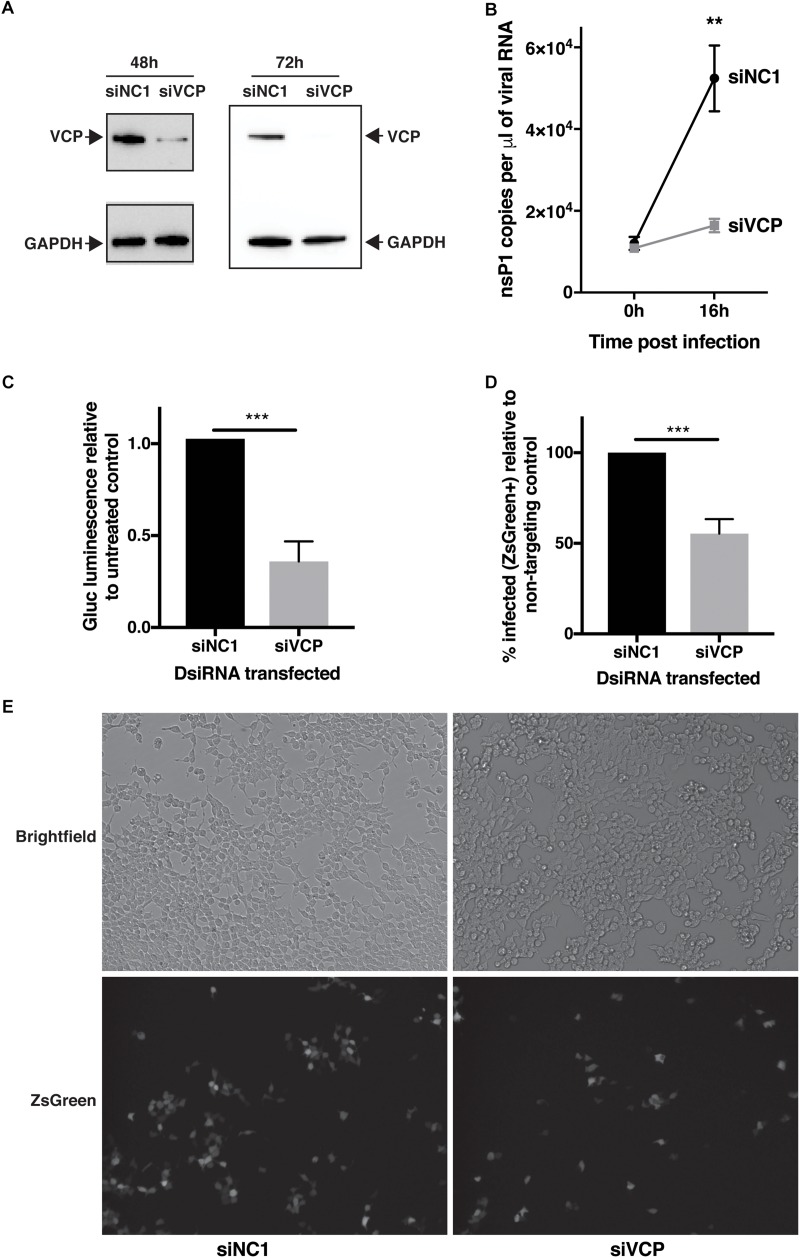
VCP knock-down affects CHIKV infection. HEK 293T cells were transfected with 10 nM of siRNA. siNC1 is a non-targeting control and siVCP is a mix of three individual siRNA targeting VCP. At 48 h post transfection, cells were infected with Gluc- or ZsGreen-tagged virus per well, inoculum was not removed, to obtain 0 hpi time point, 140 μL of supernatant was collected immediately after addition of inoculum. At 16 h post-infection, supernatant was collected or cells were imaged and harvested for flow cytometry analysis, respectively. **(A)** Representative western blot images of three independent experiment for VCP and GAPDH expression on cell lysates at 48 and 64 h post transfection. **(B)** Taqman quantification of viral RNA in the cell supernatant at 0 and 16 hpi. **(C)** *Gaussia* luciferase luminescence relative to siNC1. **(D)** % of FITC positive (infected) cells in siVCP-transfected cells relative to siNC1-transfected cells analyzed by flow cytometry. **(E)** Representative images of brightfield (auto exposure) and FITC (fixed exposure) channel on an epi-fluorescence microscope at 16 hpi. The data are presented as mean ± SD from minimum three independent experiments (two for panel **B**) and were analyzed by Mann–Whitney non-parametric two-tailed test; ^∗∗^*p* < 0.01; ^∗∗∗^*p* < 0.001.

In order to assess if VCP has a role during CHIKV infection, transfection of a mix of three siRNAs targeting VCP followed by infection with CHIKV-expressing *Gaussia* luciferase (Gluc) inserted between non-structural and structural coding regions, as an indirect marker of viral replication, was performed. Reduction of VCP protein levels was verified by western blot assays at 48 h (time of infection) and 64 h post-siRNA transfection (16 h post infection, time of supernatant collection) (Figure 1A). Knockdown of VCP significantly reduced the increase of viral RNA in the supernantant from 0 to 16 hpi (Figure 1B). Next, we validated that Gluc luminescence correlated with viral RNA and viral particle amounts in the same cell supernatants (Supplementary Figure S1), confirming that Gluc luminescence could be used as a marker of viral replication. Using Gluc, we confirmed that VCP knockdown severely impacted viral replication (Figure 1C).

In order to validate that this effect was not due to a VCP-dependant release or secretion defect of the Gluc or the viral particles, the experiment was repeated using a virus expressing a non-secreted ZsGreen reporter instead marking infected cells. In that context, VCP knockdown also significantly reduced viral infection (Figures 1D,E). Taken together, these results suggest a proviral role of VCP during CHIKV infection.

### VCP Inhibition Does Not Affect CHIKV Binding and Entry

**FIGURE 2:**
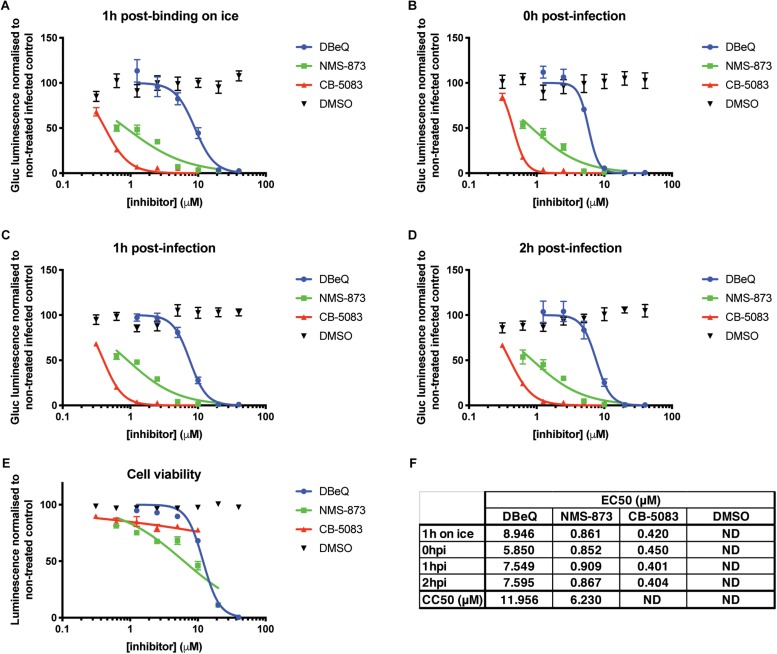
VCP chemical inhibition affects CHIKV infection. Thirty thousand HEK 293T cells per well in 96-well plates fibronectin coated were treated with VCP inhibitors CB-5083, NMS-873, DBeQ, or DMSO control at the times indicated on the panels relative to infection with 30,000 pfu of Gluc-tagged CHIKV per well. At 16 hpi, luminescence was quantified from 50 μL of supernatant. Data are presented as % luminescence compared to non-treated infected cells in *y*-axis, and as concentration of inhibitor (μM) in the *x*-axis. **(A)** Virus was allowed to bind to the cells for 1 h on ice before inoculate was removed and virus-free media with compounds were applied and cells moved back to 37°C. **(B)** Virus was applied to the cells at the same time as the compounds, removed after 1 h and virus-free media with compound was replaced on the cells. **(C,D)** Virus was applied for 1 or 2 h, respectively, after which inoculum was removed and compounds were applied. **(E)** Cytotoxicity of the compounds was assessed for each compound by CellTiter-Glo assay without infection at 16 h post treatment. **(F)** EC50 and CC50 values from panels **A–E** were calculated using the non-linear regression analysis (inhibitor) (μM) vs. normalized response variable slope function of GraphPad Prism. The data presented in panels **A–E** are mean ± SEM of three independent experiments. ND, not determined.

In order to circumvent limitations in assessing the various steps of the viral cycle affected by VCP knockdown, VCP-specific chemical inhibitors and time-of-addition assays were explored. The different chemical inhibitors assessed included DBeQ, a reversible inhibitor of VCP (Fang et al., 2015), NMS-873, a specific allosteric VCP inhibitor (Magnaghi et al., 2013), and CB-5083, an orally bioavailable active compound derived from the scaffold of DBeQ (Anderson et al., 2015). Notably, CB-5083 has been shown to be selective, specifically inhibiting the ATPase activity of the D2 domain of VCP by binding in the nucleotide binding site (Tang et al., 2019). All VCP inhibitors showed a dose-dependent effect on Gluc production with an approximate effective concentration 50 (EC50) ranging from 0.4 to 10 μM (Figure 2). Toxicity of the different inhibitors at different concentrations was verified using the Cell Titer Glo assay and showed cytotoxic concentration 50 (CC50) very close from their respective EC50 for DBeQ and NMS-873 suggesting that their inhibitory effect on viral Gluc production could be linked to the cytotoxicity effect. However, no significant toxicity for CB-5083 was observed (Figure 2E), suggesting that the inhibitory effect on CB-5083 on viral Gluc production is not due to cytotoxicity. Interestingly, the time-of-addition of chemical inhibitors relative to viral infection had no significant impact on the anti-CHIKV EC50 (Figures 2C,D,F), with CB-5083 having the lowest EC50 (0.4 < EC50 < 0.45 μM) (Figure 2E). Taken together, these results suggest an antiviral effect of VCP inhibitor CB-5083 at a post binding or entry step.

**FIGURE 3:**
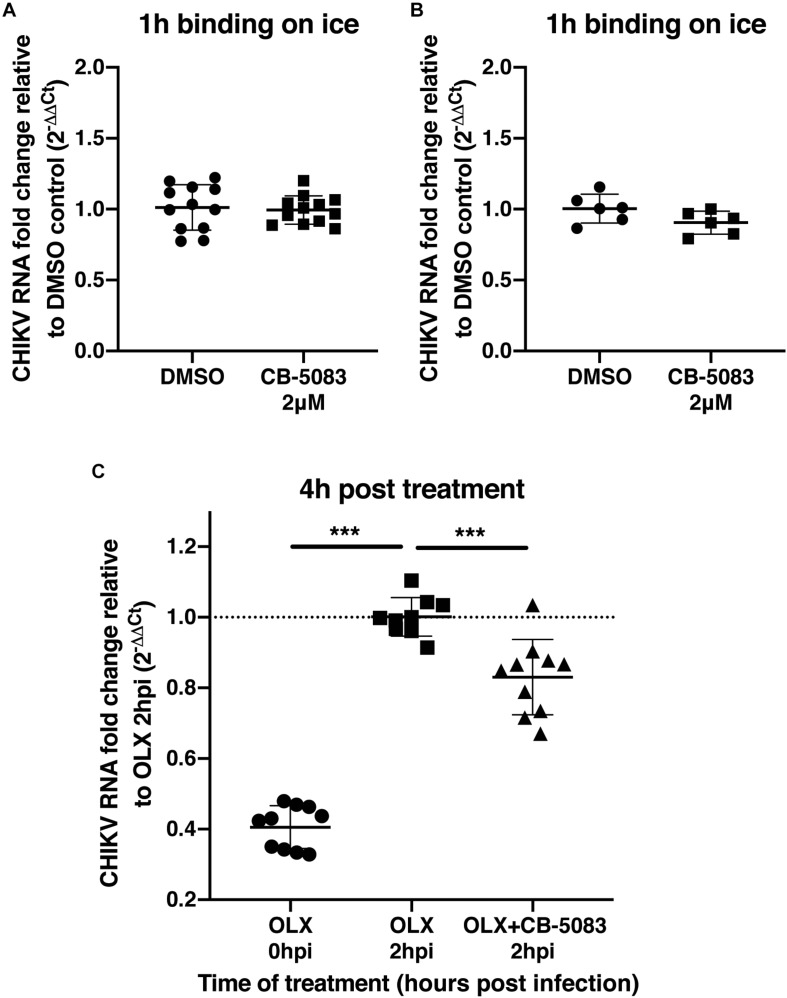
VCP is not involved in CHIKV binding or entry. **(A,B)** Two hundred thousand HEK 293T cells per well were infected at moi 10 for 1 h in DMEM media or in media with 2 μM CB-5083 on ice or at 37°C, respectively. Cells were then washed twice in PBS, collected, and RNA was extracted. **(C)** Two hundred thousand HEK293T cells per well were infected at moi 10 for 2 h in DMEM media or in media with 0.5 μM OLX. After 2 h, inoculum was removed, cells were washed twice and incubated for 4 h in media with CB-5083 at 2 μM or media with OLX (0.5 μM) and CB-5083 (2 μM) or media with OLX (0.5 μM), the cells were then washed twice, collected, and RNA was extracted. Relative CHIKV RNA fold change compared to control using 2^−ΔΔC_T_^ method. Each symbol represents an independent well, data presented are from three independent experiments for panels **A** and **C** and two independent experiments for panel **B** represented as mean ± SD and were analyzed by Mann–Whitney non-parametric two-tailed test for panel **D**; ^∗∗∗^*p* < 0.001.

To validate that VCP was not involved during CHIKV binding or entry, a binding assay was performed on ice and at 37°C for 1 h in the presence of CB-5083 or DMSO control. After the washing steps, RNA was extracted from cells and CHIKV RNA was quantified relative to GAPDH RNA for each individual sample. During binding on ice, no difference in the relative amount of CHIKV RNA was observed during VCP inhibition by CB-5083 treatment compared to the control samples (Figure 3A). Intriguingly, higher CHIKV RNA levels in the DMSO control-treated samples were observed when binding was performed at 37°C, suggesting that VCP could be involved in an early post-entry replication step. Together with the binding on ice, a lower amount of CHIKV RNA in the treated group indicates that some replication has occurred in the DMSO control, but not in the treated group.

To confirm this observation, cells were treated with an entry inhibitor, Obatoclax (OLX) (Varghese et al., 2017), or OLX together with CB-5083 at 2 hpi, and assessed for the relative amount of viral RNA at 4 h post-treatment (6 h post virus addition) (Figure 3C). VCP inhibition by CB-5083 resulted in reduced viral RNA levels compared to entry inhibition alone by OLX at 4 h post-treatment (Figure 3C). Taken together, these results show that VCP inhibition is affecting a post binding and entry replication step.

### VCP Is Essential for Viral RNA Replication

**FIGURE 4:**
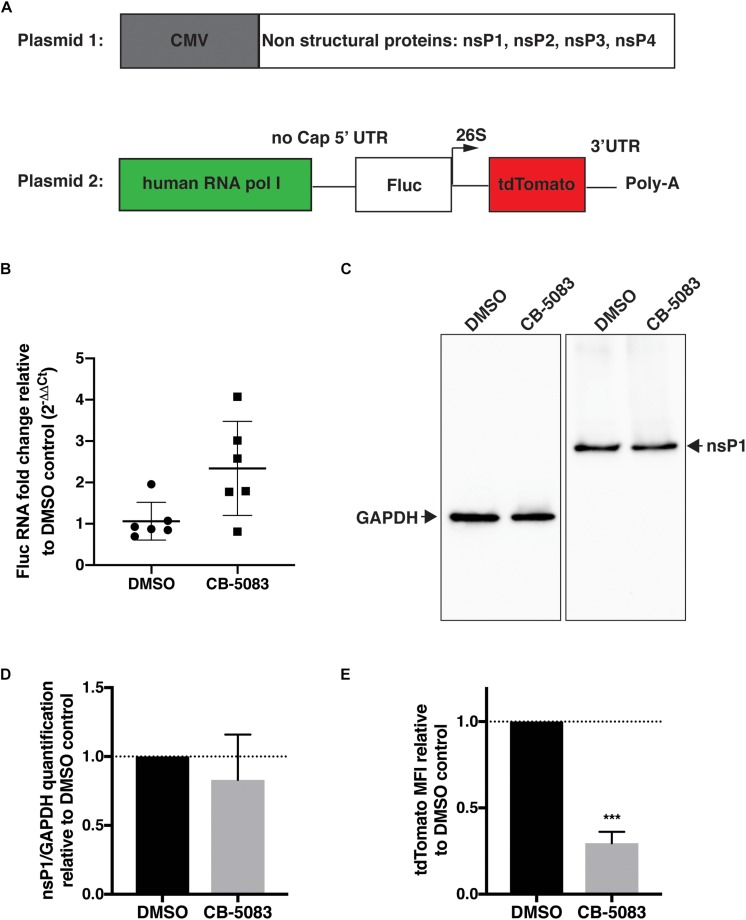
VCP is essential for CHIKV replicase activity. HEK 293T cells were co-transfected with plasmid 2 alone (panel **B**) or plasmids 1 and 2 (ratio 2:1, panels **C–E**), 5–6 h post transfection, media was refreshed with DMSO control or CB-5083 (1 μM). 16 h post media refresh, cells were collected and separated for RNA extraction or whole cell lysate and flow cytometry analysis. **(A)** Schematic representation of the CHIKV trans-replication plasmid system used. **(B)** Relative Fluc RNA fold change compared to control using 2^−ΔΔC_T_^ method. **(C)** Representative Western blot of whole cell lysates probed for GAPDH and CHIKV nsP1 proteins, respectively. **(D)** Relative quantification of nsP1 protein amount versus GAPDH for each experimental replicate. **(E)** Mean fluorescent intensity (MFI) of tdTomato assessed by flow cytometry in tdTomato positive cells. The data presented are of three independent experiments (two for panel **B**) and were analyzed by Mann–Whitney non-parametric two-tailed test; ^∗∗∗^*p* < 0.001.

To confirm that inhibition of VCP impacts viral RNA replication, a CHIKV trans-replication system was established (Figure 4A) (Utt et al., 2016; Abraham et al., 2018). In this system, the nsPs are encoded on a separate plasmid and expressed constitutively using a CMV promoter (Utt et al., 2015) (Plasmid 1, Figure 4A). Since the use of viral RNA templates generated by cellular RNA polymerase II could produce high background signals (Utt et al., 2015; Abraham et al., 2018; Bartholomeeusen et al., 2018), and the replicase of CHIKV is capable of using non-capped RNA templates (Utt et al., 2015), a human RNA polymerase I promoter was used to generate non-capped viral RNA template (Plasmid 2, Figure 4A) (Utt et al., 2019). This template codes for *Firefly* luciferase (Fluc) instead of the non-structural open-reading frame (ORF) and codes for tdTomato instead of the structural ORF (Plasmid 2, Figure 4A). These reporters would be translated after template RNA replication by the viral replicase, with minimal expression of either reporter in absence of template RNA replication. Thus, tdTomato protein would be expressed and detectable only when RNA replication occurred allowing us to assess the effect of VCP inhibitor on viral replication.

Using this system, we first verified that VCP inhibition by CB-5083 had no effect on transcription by RNA polymerase I by quantifying Fluc RNA levels relative to GAPDH in the absence of replicase proteins (plasmid 2 alone) (Figure 4B). Surprisingly, an increase in template RNA levels was found in CB-5083-treated samples, suggesting that VCP inhibition by CB-5083 could increase RNA polymerase I transcription or/and increase stability of non-capped RNA transcripts. Next, we verified that VCP inhibition did not affect expression of the nsPs from the CMV plasmid by assessing nsP1 protein levels by western blot (Figures 4C,D). Altogether, results showed that CB-5083 had no negative effect on production of CHIKV replicase proteins or template RNA. However, when the replicase and templates were co-expressed, VCP chemical inhibition drastically decreased the amount of tdTomato mean fluorescence intensity (MFI) detected per transfected cell (Figure 4E) despite the increased Fluc RNA template availability. These results confirm that VCP is a proviral factor essential during CHIKV replication at a post entry step.

### VCP Co-localizes With CHIKV nsPs

**FIGURE 5:**
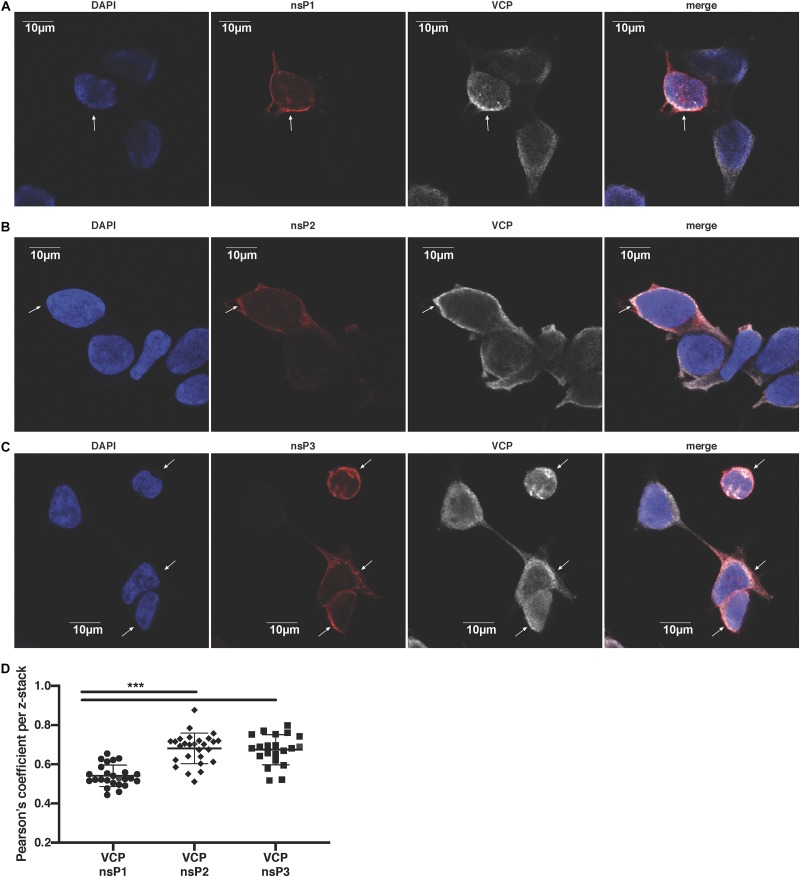
VCP localizes with CHIKV replicase components. HEK 293T cells were infected at an moi of 10, 2 hpi cells were then plated in Ibidi μ-wells microscopy chambers; 5 hours post-plating, cells were fixed, permeabilized, and co-stained in duplicate for VCP with nsP1, nsP2, or nsP3 as indicated on the figure. Cells were counterstained with DAPI before confocal acquisition of z-stacks. **(A)** Representative focal plane of VCP with nsP1. **(B)** Representative focal plane of VCP with nsP2. **(C)** Representative focal plane of VCP with nsP3. **(D)** Pearson correlation coefficient of colocalization of VCP with the respective nsPs in each z-stack analyzed with Imaris software. Results are representative of two independent experiments performed in duplicate. White arrows indicate infected cells. Correlation coefficient were analyzed by Mann–Whitney non-parametric two-tailed test; ^∗∗∗^*p* < 0.001.

We next assessed if VCP co-localizes specifically with a particular nsP. Using confocal microscopy, we observed that VCP was partially co-localizing with nsP1 (Figure 5A), nsP2 (Figure 5B), and nsP3 (Figure 5C) in virus-infected cells. Unfortunately, in this assay, nsP4 as well as dsRNA co-localization with VCP could not be assessed due to lack of suitable and species compatible antibodies, respectively. Pearson’s correlation coefficient for all the acquired images indicated that VCP co-localized more with nsP2 and nsP3 than nsP1 (Figure 5D). These results suggest that VCP could likely interact with multiple viral nsPs, or is associated with a nsP in replicase complexes in order to perform proviral functions.

### VCP Does Not Co-localize With dsRNA

**FIGURE 6:**
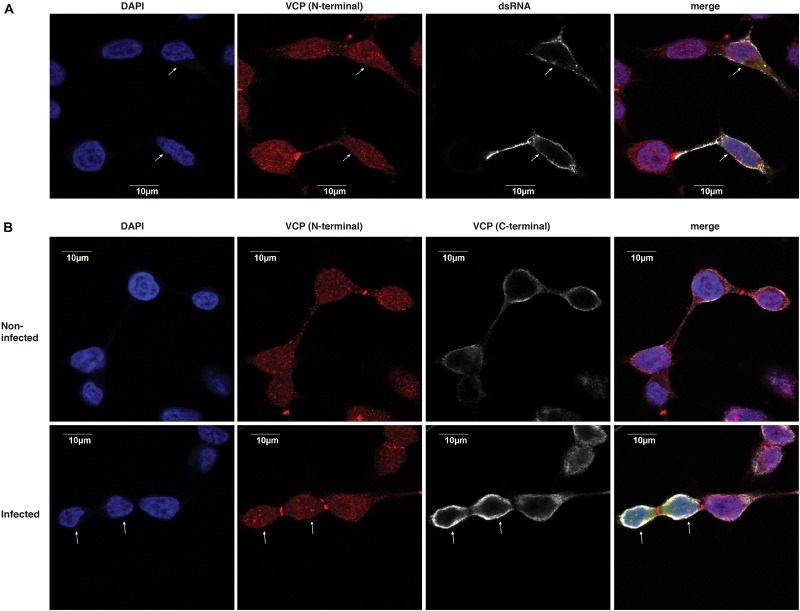
dsRNA foci do not colocalize with VCP. HEK 293T cells were infected at an moi of 10 with ZsGreen-tagged CHIKV-LR, 2 hpi cells were washed and re-plated in Ibidi μ-wells microscopy chambers. At 5 h post-plating, cells were fixed, permeabilized, and co-stained for VCP and dsRNA as indicated on the figure. Cells were counterstained with DAPI before confocal acquisition. **(A)** Representative focal plane of VCP with dsRNA staining in infected cells. **(B)** Representative focal plane of VCP (N-terminal) and (C-terminal) staining. Results are representative of two independent experiments performed in triplicates. White arrows indicate infected cells which were determined using the ZsGreen channel (shown only in the merged image).

We then assessed if VCP could localize with dsRNA replication complexes. Therefore, we assessed the performance of a polyclonal rabbit anti-VCP antibody in immunofluorescence assay which would allow co-localization with the mouse antibody recognizing dsRNA. Using this combinaition of antibodies, we did not observe co-localization between VCP and dsRNA foci (Figure 6A). Interestingly, this antibody against VCP presented a very different localization pattern than the mouse monoclonal antibody used previously in both infected and non-infected conditions (Figure 5). Since the rabbit antibody, directed against the N-terminal, gave a punctuated localization different from the pattern observed previously near the cytosolic membrane with the mouse monoclonal antibody against VCP C-terminal (Figure 5), we assessed co-localization of the two anti-VCP antibodies (Figure 6B). Signals from both antibodies did not co-localize, and localization of VCP near the plasma membrane was not observable using the N-terminal directed antibody raised in rabbit. Since the N-terminal of VCP is a site of interaction with several co-factors (Hanzelmann and Schindelin, 2017), we hypothesize that the observed absence of co-localization of dsRNA with VCP could be due to a lack of N-terminal accessibility.

### VCP Is a Proviral Factor for Geographically Different Alphaviruses

**FIGURE 7:**
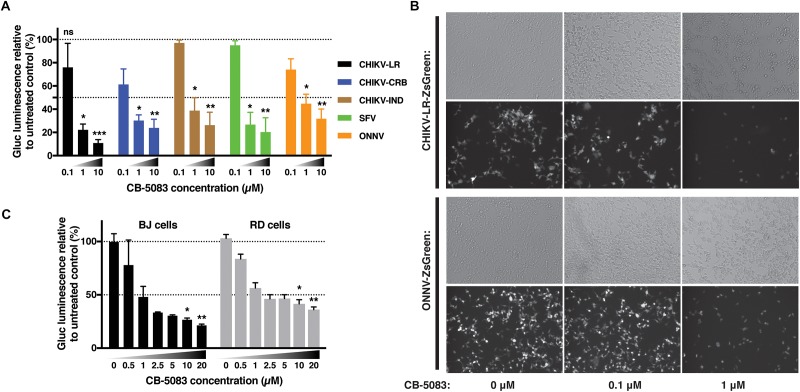
VCP is proviral for other alphaviruses and in other cell types. **(A)** Thirty thousand HEK 293T cells per well were treated with increasing concentrations of CB-5083 (0, 0.1, 1, and 10 μM) and infected by 3,000 pfu/well of the indicated viruses each tagged with Gluc, virus inoculum was not removed. At 16 hpi, luminescence in 50 μL of supernatant was assessed, results are expressed to their relative 0 μM treatment. **(B)** Representative epi-fluorescence images of 30,000 HEK 293T cells infected by 30,000 pfu of ZsGreen-tagged CHIKV-LR and ONNV for 16 h at different CB-5083 concentration (as indicated on the figure). Images are representative of three independent experiments and were acquired on brightfield (auto exposure) and FITC (fixed exposure) channels. **(C)** 5,000 RD or BJ cells per well were treated with increasing concentrations of CB-5083 (indicated on the figure) and infected by 5,000 pfu of CHIKV-LR-tagged with Gluc, virus inoculum was not removed. At 16 hpi, luminescence in 50 μL of supernatant was assessed. Data of panels **A** and **B** are representative of four and three independent experiment, respectively, presented as mean ± SD and were analyzed by Mann–Whitney non-parametric two-tailed test; ^∗^*p* < 0.05, ^∗∗^*p* < 0.01, ^∗∗∗^*p* < 0.001.

In order to assess if this effect was restricted to the CHIKV viral isolate used in the previous experiments, CB-5083 was used to assess the role of VCP during infection with different CHIKV isolates as well as the closely related ONNV, and more distantly related SFV all belonging to the SFV clade. CHIKV can be categorized into three main genotypes, the West Africa, East-Central-South-African (ECSA), and Asian genotypes. We compared the Chikungunya strain isolated during the La Reunion epidemic [La Reunion isolate LR2006-OPY1 (LR)] and an Indian (IND) strain isolated in India during the same epidemic from a patient presenting neurological complications both belonging to the ECSA genotype. We also compared a Caribbean (CRB) isolate (CNR20235) belonging to the Asian genotype, isolated during outbreaks in the CRB islands in December 2013, which we have previously shown to be less pathogenic than the LR isolate *in vitro* and *in vivo* in mouse models (Teo et al., 2015). VCP inhibition with CB-5083 showed a dose-dependent inhibition of viral Gluc production with all viruses tested (Figure 7A). A dose-dependent decrease of ZsGreen signal was also observed in cells infected with ZsGreen-tagged CHIKV or ONNV (Figure 7B). In addition, we assessed if the effect of VCP inhibition on CHIKV replication was reproducible in more relevant human cells related to cell types infected in patients: a human foreskin fibroblast cell line (BJ cells) and a muscle cells line (RD cells) (Suhrbier et al., 2012). VCP inhibition with CB-5083 resulted in dose-dependent inhibition of CHIKV in BJ and RD cells (Figure 7C).

## Discussion

In this study, we show that siRNAs targeting VCP (Figure 1) as well as chemical inhibitors (Figure 2) are able to reduce CHIKV infection and replication in HEK293T cells. Time-of-addition assays using specific chemical inhibitors of VCP suggested that the virus life cycle step involving VCP as a proviral factor would be viral RNA replication rather than binding or entry. Although the effect of VCP inhibition demonstred to be conserved across different alphaviruses (Figure 7), variations in the level of virus inhibition were observed between the different cell types and virus isolates. These variations are likely due to the calculation for each virus and cell type, where the relative luciferase is normalized against the signals from the untreated infected internal controls, where background signal comes from initial Gluc contained in the inoculum.

Despite the proviral role of VCP on CHIKV replication observed during knockdown or chemical inhibition, no increase in viral replication upon VCP or VCP domains overexpression was observed (Supplementary Figure S2), consistent with VCP being highly abundant (0.7% of cytoplasmic protein content) (Peters et al., 1990; Zeiler et al., 2012; Meyer and Weihl, 2014). Only transfection of VCP truncated of the domain 2 (ND1 mutant) showed an inhibitory effect on Gluc production. This effect is not surprising as this truncated VCP is a known dominant-negative mutant of VCP (Ye et al., 2003; van den Boom et al., 2016). These results are consistent with both siRNA and chemical inhibitors effects.

Interestingly, a genome-wide screen on insect cells had previously identified VCP as a proviral entry factor for SINV through the downregulation of its receptor NRAMP2 (Rose et al., 2011; Panda et al., 2013). However, we did not observe a role for VCP as a binding factor of CHIKV (Figures 3A,B), consistent with the fact that CHIKV and other alphaviruses binding and entry is NRAMP2 independent (Rose et al., 2011). This observation was further verified using an entry assay (Figure 3C), which showed an effect of VCP inhibition on intracellular viral levels consistent with a role of VCP during viral RNA replication. Therefore, excluding a role during viral binding or entry in our experimental setup. However, we cannot exclude that VCP inhibition prior to infection could also result in lower trafficking of viral receptor or other entry factors.

Nevertheless, the trans-replication assay further confirmed the role of VCP inhibition during viral RNA replication, independent of the stability and template availability of alphavirus nsPs (Figure 4). In addition, viral replicase components nsP1, 2, and 3 were demonstrated to co-localize partially with VCP differentially (Figure 5). This difference could be due to the fact that a significant portion of nsP1 is anchored in the plasma membrane while VCP, nsP2, and nsP3 are cytoplasmic. These results are consistent with earlier studies with SFV and HCV showing association of VCP with their replication complexes (Varjak et al., 2013; Yi et al., 2016), and the role of VCP during virus RNA replication of poliovirus, enterovirus 71, human cytomegalovirus, West Nile virus, and HCV (Arita et al., 2012; Wu et al., 2016; Yi et al., 2016; Lin et al., 2017; Phongphaew et al., 2017; Wang et al., 2017; Yi and Yuan, 2017). Interestingly, VCP was also involved in the egress of viral particles for poliovirus and Rift Valley fever virus (RVFV) (Arita et al., 2012; Brahms et al., 2017). The results on RVFV are of particular interest, where the study identified VCP as the target of FDA-approved drug Sorafenib effect on RVFV (Benedict et al., 2015; Brahms et al., 2017). The same group has also identified Sorafenib as a potential drug that reduces replication of several alphaviruses including CHIKV (Lundberg et al., 2018). It would be interesting to verify if Sorafenib inhibits alphaviruses in a VCP-dependant manner similar to RVFV.

Interestingly, we could not observe co-localization of VCP with dsRNA foci of replication complexes (Figure 6A). This is likely due to the unavailability of the VCP N-terminal for recognition with the antibody used, which is reinforced by the lack of co-localization of the VCP antibodies recognizing the C- and N-termini (Figure 6B). Importantly, the apparent re-localization of VCP with an available C-terminal at the infected cell membrane is consistent with the localization of the dsRNA foci. Further studies will be needed to assess if and how VCP is involved in the replication complexes of CHIKV and other alphaviruses.

Valosin-containing protein is an essential host protein involved in a wide range of cellular processes (Meyer and Weihl, 2014). A link between one or more of the described VCP functions and viral replication remains poorly understood. For example during infection, virus could use VCP to hijack ER or mitochondria-associated degradation, proteasome, or UPS modulating functions (Taylor and Rutter, 2011; Wolf and Stolz, 2012). Proteasome activity was reported to be essential for CHIKV replication (Karpe et al., 2016), and CHIKV can manipulate the UPS to prevent host-cell shut-off (Fros et al., 2015). Along the same line, CHIKV could use VCP modulating function on autophagy. Such mechanism could explain how CHIKV induces autophagy (Papadopoulos and Meyer, 2017) to delay cell death and increase replication (Joubert et al., 2012a, b). It is also plausible that VCP’s role in membrane rearrangement (Zhang and Wang, 2015) could be hijacked by the virus in order to form invaginated membranous structures of the replication complexes efficiently (Reid et al., 2015). This would explain VCP co-localization with CHIKV nsPs (Figure 5), and its presence in replication complexes of HCV and SFV (Varjak et al., 2013; Yi et al., 2016).

Similarly, VCP is important for lipid droplets metabolism (Olzmann et al., 2013), a site at which CHIKV nsP3 was also shown to associate (Remenyi et al., 2017). Moreover, lipid droplets metabolism was also shown to be important for flavivirus replication (Zhang et al., 2017), in which VCP function as a proviral host factor for virus replication (Phongphaew et al., 2017). VCP unfolding of aggregated proteins could also be a necessary function for viruses, for example during HCV infection, VCP prevents NS5A from being over aggregated and non-functional (Yi and Yuan, 2017). Other cellular functions of VCP, such as NF-κB activation (Schweitzer et al., 2016), stalled ribosome turnover (Verma et al., 2013), full capacity of autophagy and lysosomal system (Papadopoulos and Meyer, 2017), are cellular functions and pathways that could possess pro- or anti-viral functions. A link between virus interaction with VCP and modulation of these functions could also present avenues for future investigations.

To conclude, we have shown that VCP, a highly conserved and connected protein with functions in multiple cellular processes has proviral functions during CHIKV replication. This effect extends to several other alphaviruses and understanding the exact role played by VCP during virus replication could shed light on a highly conserved viral subversion mechanism of cellular machineries via the multi-purpose protein VCP. This accumulating evidence suggest that understanding of such mechanism could open the way for designing new treatment strategies effective against a wide range of viruses by targeting a conserved VCP proviral role.

## Materials and Methods

### Cell Lines

Human embryonic kidney HEK293T cells (ATCC-CRL-3216), human foreskin fibroblast BJ cells (ATCC-CRL-2522), human muscle RD cells (ATCC-CCL-136), and African green monkey kidney Vero E6 cells (ATCC-CRL-1586) were grown in Dulbecco’s modified Eagle medium (DMEM) supplemented with 10% fetal bovine serum (FBS, Gibco); and for BJ cells supplemented with 1× Penicillin–Streptomycin (Gibco). All cells were maintained at 37°C with 5% CO_2_.

### Viruses and Strains

Chikungunya virus infectious clone plasmids containing a duplicated subgenomic promoter and insertion of Gluc (*Gaussia princeps* luciferase) or ZsGreen (*Anthozoa* reef coral fluorescent protein) between non-structural and structural regions were constructed for isolates of La Reunion (LR2006 OPY1), the CRB region (CNR20235), and the IND region. The CHIKV IND strain was isolated on BHK cells from the cerebral spinal fluid of an IND patient during the 2010 epidemic (gift from Dr. Ooi Eng Eong, DUKE-NUS Graduate Medical School, Singapore) and subsequently passaged on Vero E6 cells. Infectious clones for ONNV (Bessaud et al., 2006) and SFV6 (Ferguson et al., 2015) were constructed in a similar fashion except that for SFV6 CMV promoter was placed upstream of viral sequence and duplicated subgenomic promoter and reporter gene were placed downstream of structural region. CHIKV and ONNV were produced using *in vitro* transcribed viral RNA from *Not*I (NEB) linearized plasmids using SP6 mMessage mMachine (Ambion). Viral RNA was then transfected in Vero E6 cells using Lipofectamine 2000 (Invitrogen) following manufacturer’s recommendation. SFV6 was produced by transfection of plasmid containing infectious cDNA in HEK293T cells. Viruses were passaged once or twice in Vero E6 cells to obtain a sufficient titer. Virus stock titration was performed by standard plaque assay on Vero E6 cells in a 6-well plate format.

### CHIKV Trans-Replication System

The CHIKV trans-replication system previously published (Utt et al., 2015, 2016, 2019) was modified by insertion of sequence encoding for a fluorescent tdTomato reporter in the template and used a human RNA polymerase I promoter instead of T7 promoter for synthesis of non-capped RNAs in transfected cells (Abraham et al., 2018). Briefly, we substituted the T7 promoter and terminator in the T7-Fluc-Gluc template (Utt et al., 2015) to human RNA polymerase I promoter and terminator (Abraham et al., 2018); the sequence corresponding to the 5′-end of CHIKV genome was placed to the start site of promoter. The Gluc reporter gene was substituted by sequence encoding tdTomato using restriction-based cloning; obtained construct was designated Pol1HS-Fluc-tdTomato. The detailed description of properties of such a system were published elsewhere (Utt et al., 2019). For verification of template generation from plasmid 2, HEK293T cells were plated at a density of 150,000 cells per well in a 12-well plate. A total of 333 ng of plasmid 2 per well was transfected using Lipofectamine 2000 (Invitrogen) following manufacturer recommendations. For the full trans-replication assay, HEK293T cells were plated at a density of 400,000 cells per well in a six-well plate. Two micrograms of plasmid 1 and 1 μg of plasmid 2 (Figure 4A) per well were co-transfected using Lipofectamine 2000. At 5–6 h post-transfection (hpt), media was refreshed for media with 1 μM CB-5083 or DMSO control. At 16 h post-media refresh cells were collected and RNA was extracted using Nucleospin RNA kit (Macherey-Nagel) following manufacturer protocol or cells were collected and half was lyzed in RIPA buffer for immunoblotting and the other half was processed for flow cytometry.

### siRNA Knock Down Assays

Predesigned dicer substrate siRNAs against VCP (hs.Ri.VCP.13) were purchased from IDT [TriFECTa kit containing non-targeting control 1 (NC1) and TYE593 labeled control siRNA]. HEK293T cells were plated either in 12-well plate or 96-well plates at a density of 100,000 or 10,000 cells/well and siRNA was transfected at 10 nM final concentration using a ratio of 1 μL of Lipofectamine 2000 for 5 pmol of DsiRNA. 24 hpt of TYE593-labeled control siRNA indicated >98% of transfection efficiency (not shown). At 48 h post siRNA transfection, cells were infected overnight with Gluc- or ZsGreen-tagged CHIKV, followed by RT-qPCR, plaque assay, or luciferase assay of supernatant, or flow cytometry analysis of cells, respectively. VCP expression in cell lysate was verified by western blot at 48 hpt and time of supernatant collection.

### Antiviral Assay

DBeQ, NMS-873, and CB-5083 were purchased from ApexBio Technology LLC and resuspended in DMSO at a final concentration of 50 mM, OLX (Selleckchem) was resuspended in DMSO at a final concentration of 100 mM. HEK293T cells were plated at 30,000 cells/well in a 96-well format (on fibronectin-coated plates for Figure 2). After adhesion, Gluc-expressing viruses at a MOI of 0.1 (3,000 pfu) or MOI 1 for Figure 2, and serially diluted compounds were added to the cells. For Figure 2, inoculums were removed as the times indicated in the legends before diluted compounds were added back on the cells. After 16 h of incubation, 50 μL of supernatant was used to determine luminescence as a proxy for viral replication. Alternatively, ZsGreen-expressing viruses were used at MOI 1.

### Binding and Entry Assay

HEK293T cells were plated at a density of 200,000 cells per well in 24-well plates. After adhesion, cells were infected at a MOI of 10 in a volume of 150 μL of DMEM media with CB-5083 at 2 μM (and DMSO control) on ice or at 37°C with 5% CO_2_ for 1 h. Alternatively, cells were infected for 2 h in complete or in complete media with 0.5 μM OLX, inoculum was removed and cells were washed with PBS. Complete media containing 0.5 μM OLX or 0.5 μM OLX with 2 μM CB-5083 was then applied to the cells for 4 h. Cells were then washed, collected in PBS, and RNA was extracted using Nucleospin RNA kit (Macherey-Nagel) following manufacturer protocol.

### qRT-PCR

For relative quantitation, eluted RNA were quantified using a NanoDrop 1000 Spectrophotometer (Thermo Scientific), diluted to a concentration of approximately 10 ng/μl, and subjected to qRT-PCR using a QuantiFast SYBR green RT-PCR kit (Qiagen) according to the manufacturer’s protocol. qRT-PCR was performed with an Applied Biosystems (ABI) 7900HT Fast Real-Time PCR system in 364-well plate format, with the following conditions: (a) reverse transcription step (50°C for 10 min; 1 cycle); (b) PCR initial activation step (95°C for 5 min; 1 cycle); and (c) 2-step cycling (95°C for 10 s, followed by 60°C for 30 s; 40 cycles). The ΔCt are *C*_T_ values of CHIKV E1 encoding RNA (or Fluc RNA) – GAPDH (housekeeping gene) were determined within samples. The fold change relative to control samples was determined using the ΔΔCt method using Microsoft Excel 2016. Briefly, ΔΔ*C*_T_ was calculated as Δ*C*_T__[__sample__]_ – Δ*C*_T__[__control sample__]_. The fold change for CHIKV E1 RNA between the sample and control was calculated as 2^-^^ΔΔC_T_^.

For viral RNA quantification in supernatants, 140 μL of infected cell supernatant was extracted using QIAmp Viral RNA kit (Qiagen), according to the manufacturer’s protocol. CHIKV viral genome copies were quantified by Taqman RT-qPCR targeting viral RNA negative sense at the nsP1 region, as previously described (Kam et al., 2012; Teo et al., 2013; Carissimo et al., 2019). Briefly, qRT-PCR was performed with an Applied Biosystems (ABI) 7900HT Fast Real-Time PCR system in 364-well plate format, with the following conditions: (a) reverse transcription step (50°C for 30 min; 1 cycle); (b) PCR initial activation step (95°C for 15 min; 1 cycle); and (c) 2-step cycling (94°C for 15 s, followed by 55°C 1 min; 45 cycles). Viral RNA copy numbers were extrapolated using the SDS software and the standard curve of serially diluted CHIKV nsP1 RNA *in vitro* produced transcripts ranging from 10 to 10^9^ copy per μL. Primers used in this study are given in Table 1.

**TABLE 1:** Primers used in this study given in 5′–3′ orientation.

**Name**	**Sequence 5′–3′ orientation**
CHIKV E1 F	AAGCTCCGCGTCCTTTACCAAG
CHIKV E1 R	CCAAATTGTCCTGGTCTTCCT
Template Fluc F	GTGGTGTGCAGCGAGAATAG
Template Fluc R	CGCTCGTTGTAGATGTCGTTAG
GAPDH F	CCACATCGCTCAGACACCAT
GAPDH R	GGCAACAATATCCACTTTACCAGAGT
CHIKV nsP1 F	GGCAGTATCGTGAATTCGATGCGACACGGA GACGCCAACATT
CHIKV nsP1 R	AATAAATCATAAGTCTGCTCTCTGTCTACATGA
CHIKV nsP1 probe	[6FAM]TGCTTACACACAGACGT[TAM]
VCP-FL amplification F	TATAAGCTTATGGCTTCTGGAGCCGATTCAA
VCP-FL amplification R	TGCGGCCGCTTAGCCATACAGGTCATCATCATT
VCP-N amplification F	TATAAGCTTATGGCTTCTGGAGCCGATTCAA
VCP-N amplification R	TGCGGCCGCTTACAAGGACTCTTCCTCATCCTC
VCP-ND1 amplification F	TATAAGCTTATGGCTTCTGGAGCCGATTCAA
VCP-ND1 amplification R	TGCGGCCGCTTACTCTACCACGGTTTCCCGCA
D2 from FL F (SDM)	GTGCCACAGGTAACCTGG
D2 from FL R (SDM)	AAGCTTGTCATCGTCATCC

*Red indicates restriction sites. F, forward; R, reverse; SDM, site-directed mutagenesis.*

### *Gaussia* Luciferase Luminescence Assay

Luminescence was assessed using the GloMax Multi-detection system (Promega) at 10-s integration time with a 2 s delay after injection 50 μL of 20 μM XenoLight RediJect coelenterazine h (PerkinElmer) diluted in PBS (Gibco) with 5 mM NaCl (Ambion). Results are presented as a percentage of luminescence of treated wells relative to luminescence of control treated wells.

### Cell Cytotoxicity Assays

Cell viability assays were carried out with the CellTiter-Glo luminescent cell viability assay (Promega), which serves as a readout for cellular ATP levels, as previously described (Ching et al., 2015). Briefly, serial dilutions of DBeQ, NMS-873, and CB-5083 were added to HEK-293T seeded on 96-well fibronectin-coated plates. After 16 h of incubation, cell viability assays using CellTiter-Glo Reagent (Promega) were performed as per the manufacturer’s protocol. After 1 h of incubation at room temperature, luminescent emissions were detected by the GloMax Multi-detection system (Promega).

### Flow Cytometry

For HEK 293T, cells from technical replicates were resuspended in their culture media, pooled, washed with PBS, fixed for 5 min in BD FACS Lysing Solution (BD Biosciences), and then acquired on LSR Fortessa flow cytometer (BD Biosciences) on the FITC or PE channel. Flow cytometry analysis was performed on minimum 30,000 cells per replicate with Flowjo 10.1 (Flowjo LLC) and single cells were assessed for FITC or PE positive signal. Data are expressed as % of infected cells in treated condition relative to % infected cells in control condition or as mean of PE intensity of PE positive cells of treated sample relative to mean of PE intensity of PE positive cells of control sample. FCS files are available as Supplementary File S1.

### Epi-Fluorescence Microscopy

Microscopy images were acquired with a 10× zoom on an Olympus epi-fluorescent microscope (IX81) at a fixed exposure time for the FITC filter or auto exposure for the brightfield filter with CellSens Dimension software (Olympus). Tiff images were opened in Photoshop (Adobe) and light and contrast were adjusted using the exact same parameters for paired images and converted to jpeg format for inclusion on the figures. Unmodified tiff images are available on request.

### Plasmid Contruction

Valosin-containing protein and VCP domains N and ND1 were amplified by PCR from HEK293T cDNA using the primers indicated in Table 1 and Q5 HiFi polymerase (NEB) following manufacturer recommendations. PCR were ligated in p3XFLAG-CMV^TM^-10 (Sigma) using *Not*I and *Hin*dIII restriction digestions (NEB) to generate flag-tagged VCP full length expression plasmid (FL) and mutants N and ND1. D2 domain was generated using site-directed mutagenesis (SDM) kit from NEB following manufacturer recommendations and primers given in Table 1. All plasmids were verified by sanger sequencing before use and correct expression was confirmed by western blot using monoclonal mouse anti-flag M2 (Sigma, ref: F3165) (not shown).

### Western Blotting

Cells were dislodged from the plates in PBS, pelleted by centrifugation for 5 min at 400 × *g*, and lysed in RIPA buffer (Invitrogen) and then sample were mixed 1:1 (v/v) with 2× Laemmli buffer (Bio-Rad) with 5% β-mercaptoethanol (Sigma), and heated for 10 min at 95°C. Samples were then migrated on 4–15% TGX mini-protean gels (Bio-Rad) and transferred to nitrocellulose membrane (Bio-Rad) using a semi-dry system and Bjerrum Schafer-Nielsen Buffer [48 mM UltraPure Tris (Invitrogen), 38 mM Glycine (Bio-Rad), 20% (v/v) EMSURE Methanol (EMD Millipore)]. Membranes were then blocked in 5% (wt/v) non-fat milk powder (Nacalai Tesque) in 1× Tris Buffer Saline (1st Base) with Tween 0.1% (v/v) (Sigma) for 1 h before staining. Antibody used for staining were mouse monoclonal anti-human VCP (clone 4G9, Biolegend), mouse monoclonal anti-human GAPDH (clone FF26A/F9, Biolegend), anti-CHIKV nsP1 affinity purified IgGs produced in rabbit (in-house), and goat anti-mouse and goat anti-rabbit antibodies coupled to HRP were purchased from Santa Cruz Biotechnology (sc-2004 and sc-2005). Proteins were revealed using WesternBright ECL HRP substrate (Advansta), signal was acquired on a ChemiDoc screen touch model 2017 (Bio-Rad), and non-saturated images were quantified using ImageLab 2.3 software (Bio-Rad). Raw images of western blot are available on request.

### Immunofluorescence Assay

HEK293T cells were infected in 6-well plates for 2 h, washed, and trypsinized before replating in complete media on ibidi μ-slide 8-well plates coated with fibronectin. At 5 h post-replating, cells were washed twice with PBS and fixed in 4% paraformaldehyde-PBS solution for 10 min slides were then washed three times in PBS and incubated 30 min in permabilization and blocking buffer (PBS with 0.1% Tween 20, 0.1% Triton X-100, 3% BSA, and 5% FBS). Cells were then incubated 2 h at room temperature with mouse anti-VCP (1:1000 dilution, Santa Cruz Biotechnology, sc-57492, monoclonal against C-terminal), rabbit anti-VCP (1:500 dilution, Thermofisher, PA522257), mouse monoclonal anti-dsRNA (3 μg/mL dilution, Scicons, J2-1820), and rabbit anti-nsP1 or nsP2 or nsP3 (in-house) at a dilution of 1:1000. Slides were then washed four times with PBS 0.1% Tween 20 and incubated for 1 h with anti-rabbit AF555 and anti-mouse AF647 (Thermofisher A21428, A21236) at 1:2000 dilution. Slides were then washed four times in PBS 0.1% Tween 20, washed three times with PBS and Prolong Gold antifade reagent with DAPI (molecular PROBES, P36935) was added to the wells. Images were acquired on an Olympus microscope (IX81) equipped with a confocal system (FV-1000) using the FV10-ASW version 04.02.02.09. Images were analyzed with Imaris version 9.2 (Bitplane) for Pearson’s correlation coefficient for co-localization between AF555 and AF647 channels using the automatic thresholding function. Confocal images presented on the figures were exported as tiff files using the FV10-ASW software and incorporated in the figure without modifications.

### Statistical Analysis

Statistical analyses were performed using GraphPad Prism versions 7.0–8.1 for macOS (GraphPad Software), using statistical tests as mentioned in the figure legends. *P*-values considered statistically significant are represented with ^∗^ for *p* < 0.05, ^∗∗^ for *p* < 0.01, and ^∗∗∗^ for *p* < 0.001.

## Data Availability Statement

The datasets generated for this study are available on request to the corresponding author.

## Author Contributions

GC conceived and designed the experiments and analyzed the data. GC, Y-HC, AU, FA, and T-KC performed the experiments. GC, AM, and LN wrote the manuscript. AU and AM contributed reagents and materials. All authors reviewed and approved the final manuscript.

## Conflict of Interest

The authors declare that the research was conducted in the absence of any commercial or financial relationships that could be construed as a potential conflict of interest.

## Supplementary Material

FIGURE S1 Viral RNA quantification, plaque assay, and luciferase luminescence show similar results. A hundred thousand HEK 293T cells per well were transfected with siRNA at 10 nM for 48 h. Cells were then infected with 10,000 pfu of Gluc-tagged CHIKV-LR. **(A)** At 16 hpi, supernatant was collected from each well for viral RNA RT-qPCR, plaque assay and luminescence reading. **(B)** Spearman correlations of the different readings for the results presented in panel **A**. Results are from two independent experiment with 2–3 well per condition in each experiment.

FIGURE S2 **(A)** The schematic representation of flag-tagged VCP and VCP domain constructs. **(B)** HEK293T were plated in 96-well format at 30,000 cells per well. At 1 day post-plating, cells were transfected with 100 ng per well of indicated VCP constructs or empty plasmid control. At 1 day post-transfection, cells were infected using 3,000 pfu per well of Gluc-tagged CHIKV and incubated for 16 h. At which time, 50 μL of supernatant was collected for luciferase assay. Data of panel **B** are representative of four independent experiment presented as mean ± SD and analyzed by Mann–Whitney non-parametric two-tailed test; ^∗^*p* < 0.05.

FILE S1 Flow cytometry fcs files generated for Figures 1, 4.

## References

[B1] Abraham R. Hauer D. McPherson R. L. Utt A. Kirby I. T. Cohen M. S. (2018). ADP-ribosyl-binding and hydrolase activities of the alphavirus nsP3 macrodomain are critical for initiation of virus replication. *Proc. Natl. Acad. Sci. U.S.A.* 115 E10457–E10466. 10.1073/pnas.1812130115 30322911 PMC6217424

[B2] Anderson D. J. Le Moigne R. Djakovic S. Kumar B. Rice J. Wong S. (2015). Targeting the AAA ATPase p97 as an approach to treat cancer through disruption of protein homeostasis. *Cancer Cell* 28 653–665. 10.1016/j.ccell.2015.10.002 26555175 PMC4941640

[B3] Arita M. Wakita T. Shimizu H. (2012). Valosin-containing protein (VCP/p97) is required for poliovirus replication and is involved in cellular protein secretion pathway in poliovirus infection. *J. Virol.* 86 5541–5553. 10.1128/JVI.00114-12 22379090 PMC3347272

[B4] Barthelme D. Sauer R. T. (2016). Origin and functional evolution of the cdc48/p97/vcp aaa+ protein unfolding and remodeling machine. *J. Mol. Biol.* 428(9 Pt B), 1861–1869. 10.1016/j.jmb.2015.11.015 26608813 PMC4860136

[B5] Bartholomeeusen K. Utt A. Coppens S. Rausalu K. Vereecken K. Arien K. K. (2018). A Chikungunya virus trans-replicase system reveals the importance of delayed non-structural polyprotein processing for efficient replication complex formation in mosquito cells. *J. Virol.* 92:e00152-e18. 10.1128/JVI.00152-18 PMC6026725 29695432

[B6] Benedict A. Bansal N. Senina S. Hooper I. Lundberg L. de la Fuente C. (2015). Repurposing FDA-approved drugs as therapeutics to treat Rift Valley fever virus infection. *Front. Microbiol.* 6:676. 10.3389/fmicb.2015.00676 26217313 PMC4495339

[B7] Bessaud M. Peyrefitte C. N. Pastorino B. A. Gravier P. Tock F. Boete F. (2006). O’nyong-nyong virus. *Chad. Emerg. Infect. Dis.* 12 1248–1250. 16965706 10.3201/eid1208.060199 PMC3291225

[B8] Brahms A. Mudhasani R. Pinkham C. Kota K. Nasar F. Zamani R. (2017). Sorafenib impedes rift valley fever virus egress by inhibiting valosin-containing protein function in the cellular secretory pathway. *J. Virol.* 91:e00968-e17. 10.1128/JVI.00968-17 28794043 PMC5640838

[B9] Carissimo G. Teo T. H. Chan Y. H. Lee C. Y. Lee B. Torres-Ruesta A. (2019). Viperin controls chikungunya virus-specific pathogenic T cell IFNgamma Th1 stimulation in mice. *Life Sci. Alliance* 2:e201900298. 10.26508/lsa.201900298 30665948 PMC6342136

[B10] Ching K. C. Kam Y. W. Merits A. Ng L. F. Chai C. L. (2015). Trisubstituted thieno[3,2-b]pyrrole 5-carboxamides as potent inhibitors of alphaviruses. *J. Med. Chem.* 58 9196–9213. 10.1021/acs.jmedchem.5b01047 26540338

[B11] Erzberger J. P. Berger J. M. (2006). Evolutionary relationships and structural mechanisms of AAA+ proteins. *Annu. Rev. Biophys. Biomol. Struct.* 35 93–114. 10.1146/annurev.biophys.35.040405.101933 16689629

[B12] Fang C. J. Gui L. Zhang X. Moen D. R. Li K. Frankowski K. J. (2015). Evaluating p97 inhibitor analogues for their domain selectivity and potency against the p97-p47 complex. *ChemMedChem* 10 52–56. 10.1002/cmdc.201402420 25377500 PMC4280364

[B13] Ferguson M. C. Saul S. Fragkoudis R. Weisheit S. Cox J. Patabendige A. (2015). Ability of the encephalitic arbovirus semliki forest virus to cross the blood-brain barrier is determined by the charge of the E2 glycoprotein. *J. Virol.* 89 7536–7549. 10.1128/JVI.03645-14 25972559 PMC4505677

[B14] Fros J. J. Major L. D. Scholte F. E. M. Gardner J. van Hemert M. J. Suhrbier A. (2015). Chikungunya virus non-structural protein 2-mediated host shut-off disables the unfolded protein response. *J. Gen. Virol.* 96(Pt 3), 580–589. 10.1099/vir.0.071845-0 25395592

[B15] Hanzelmann P. Schindelin H. (2017). The interplay of cofactor interactions and post-translational modifications in the regulation of the AAA+ ATPase p97. *Front. Mol. Biosci.* 4:21. 10.3389/fmolb.2017.00021 28451587 PMC5389986

[B16] Joubert P. E. Werneke S. de la Calle C. Guivel-Benhassine F. Giodini A. Peduto L. (2012a). Chikungunya-induced cell death is limited by ER and oxidative stress-induced autophagy. *Autophagy* 8 1261–1263. 10.4161/auto.20751 22836517

[B17] Joubert P. E. Werneke S. W. de la Calle C. Guivel-Benhassine F. Giodini A. Peduto L. (2012b). Chikungunya virus-induced autophagy delays caspase-dependent cell death. *J. Exp. Med.* 209 1029–1047. 10.1084/jem.20110996 22508836 PMC3348111

[B18] Kam Y. W. Lum F. M. Teo T. H. Lee W. W. Simarmata D. Harjanto S. (2012). Early neutralizing IgG response to Chikungunya virus in infected patients targets a dominant linear epitope on the E2 glycoprotein. *EMBO Mol. Med.* 4 330–343. 10.1002/emmm.201200213 22389221 PMC3376860

[B19] Karpe Y. A. Pingale K. D. Kanade G. D. (2016). Activities of proteasome and m-calpain are essential for Chikungunya virus replication. *Virus Genes* 52 716–721. 10.1007/s11262-016-1355-5 27206501 PMC7088676

[B20] Lin Y. T. Prendergast J. Grey F. (2017). The host ubiquitin-dependent segregase VCP/p97 is required for the onset of human cytomegalovirus replication. *PLoS Pathog.* 13:e1006329. 10.1371/journal.ppat.1006329 28494016 PMC5426786

[B21] Lundberg L. Brahms A. Hooper I. Carey B. Lin S. C. Dahal B. (2018). Repurposed FDA-Approved drug sorafenib reduces replication of Venezuelan equine encephalitis virus and other alphaviruses. *Antiviral Res.* 157 57–67. 10.1016/j.antiviral.2018.07.005 29981794

[B22] Lyupina Y. V. Erokhov P. A. Kravchuk O. I. Finoshin A. D. Abaturova S. B. Orlova O. V. (2018). Essential function of VCP/p97 in infection cycle of the nucleopolyhedrovirus AcMNPV in Spodoptera frugiperda Sf9 cells. *Virus Res.* 253 68–76. 10.1016/j.virusres.2018.06.001 29890203

[B23] Magnaghi P. D’Alessio R. Valsasina B. Avanzi N. Rizzi S. Asa D. (2013). Covalent and allosteric inhibitors of the ATPase VCP/p97 induce cancer cell death. *Nat. Chem. Biol.* 9 548–556. 10.1038/nchembio.1313 23892893

[B24] Meyer H. Bug M. Bremer S. (2012). Emerging functions of the VCP/p97 AAA-ATPase in the ubiquitin system. *Nat. Chem. Biol.* 14 117–123. 10.1038/ncb2407 22298039

[B25] Meyer H. Weihl C. C. (2014). The VCP/p97 system at a glance: connecting cellular function to disease pathogenesis. *J. Cell Sci.* 127(Pt 18), 3877–3883. 10.1242/jcs.093831 25146396 PMC4163641

[B26] Olzmann J. A. Richter C. M. Kopito R. R. (2013). Spatial regulation of UBXD8 and p97/VCP controls ATGL-mediated lipid droplet turnover. *Proc. Natl. Acad. Sci. U.S.A.* 110 1345–1350. 10.1073/pnas.1213738110 23297223 PMC3557085

[B27] Panda D. Rose P. P. Hanna S. L. Gold B. Hopkins K. C. Lyde R. B. (2013). Genome-wide RNAi screen identifies SEC61A and VCP as conserved regulators of Sindbis virus entry. *Cell Rep.* 5 1737–1748. 10.1016/j.celrep.2013.11.028 24332855 PMC3920290

[B28] Papadopoulos C. Meyer H. (2017). Detection and clearance of damaged lysosomes by the endo-lysosomal damage response and lysophagy. *Curr. Biol.* 27 R1330–R1341. 10.1016/j.cub.2017.11.012 29257971

[B29] Peters J. M. Walsh M. J. Franke W. W. (1990). An abundant and ubiquitous homo-oligomeric ring-shaped ATPase particle related to the putative vesicle fusion proteins Sec18p and NSF. *EMBO J.* 9 1757–1767. 10.1002/j.1460-2075.1990.tb08300.x 2140770 PMC551880

[B30] Phongphaew W. Kobayashi S. Sasaki M. Carr M. Hall W. W. Orba Y. (2017). Valosin-containing protein (VCP/p97) plays a role in the replication of West Nile virus. *Virus Res.* 228 114–123. 10.1016/j.virusres.2016.11.029 27914931 PMC7114552

[B31] Ramadan K. Halder S. Wiseman K. Vaz B. (2017). Strategic role of the ubiquitin-dependent segregase p97 (VCP or Cdc48) in DNA replication. *Chromosoma* 126 17–32. 10.1007/s00412-016-0587-4 27086594

[B32] Reid C. R. Airo A. M. Hobman T. C. (2015). The virus-host interplay: biogenesis of +RNA replication complexes. *Viruses* 7 4385–4413. 10.3390/v7082825 26287230 PMC4576186

[B33] Remenyi R. Roberts G. C. Zothner C. Merits A. Harris M. (2017). SNAP-tagged Chikungunya virus replicons improve visualisation of non-structural protein 3 by fluorescence microscopy. *Sci. Rep.* 7:5682. 10.1038/s41598-017-05820-0 28720784 PMC5515888

[B34] Rose P. P. Hanna S. L. Spiridigliozzi A. Wannissorn N. Beiting D. P. Ross S. R. (2011). Natural resistance-associated macrophage protein is a cellular receptor for sindbis virus in both insect and mammalian hosts. *Cell Host Microbe.* 10 97–104. 10.1016/j.chom.2011.06.009 21843867 PMC3164510

[B35] Schweitzer K. Pralow A. Naumann M. (2016). p97/VCP promotes Cullin-RING-ubiquitin-ligase/proteasome-dependent degradation of IkappaBalpha and the preceding liberation of RelA from ubiquitinated IkappaBalpha. *J. Cell Mol. Med.* 20 58–70. 10.1111/jcmm.12702 26463447 PMC4717852

[B36] Suhrbier A. Jaffar-Bandjee M. C. Gasque P. (2012). Arthritogenic alphaviruses–an overview. *Nat. Rev. Rheumatol.* 2012 420–429. 10.1038/nrrheum.2012.64 22565316

[B37] Tang W. K. Odzorig T. Jin W. Xia D. (2019). Structural basis of p97 inhibition by the site-selective anticancer compound CB-5083. *Mol. Pharmacol.* 95 286–293. 10.1124/mol.118.114256 30591537 PMC6355941

[B38] Taylor E. B. Rutter J. (2011). Mitochondrial quality control by the ubiquitin-proteasome system. *Biochem. Soc. Trans.* 39 1509–1513. 10.1042/BST0391509 21936843

[B39] Teo T. H. Her Z. Tan J. J. Lum F. M. Lee W. W. Chan Y. H. (2015). Caribbean and La Reunion Chikungunya virus isolates differ in their capacity to induce proinflammatory Th1 and NK Cell responses and acute joint pathology. *J. Virol.* 89 7955–7969. 10.1128/JVI.00909-15 25995257 PMC4505608

[B40] Teo T. H. Lum F. M. Claser C. Lulla V. Lulla A. Merits A. (2013). A pathogenic role for CD4+ T cells during Chikungunya virus infection in mice. *J. Immunol.* 190 259–269. 10.4049/jimmunol.1202177 23209328

[B41] Torrecilla I. Oehler J. Ramadan K. (2017). The role of ubiquitin-dependent segregase p97 (VCP or Cdc48) in chromatin dynamics after DNA double strand breaks. *Philos. Trans. R. Soc. Lond. B Biol. Sci.* 372:20160282. 10.1098/rstb.2016.0282 28847819 PMC5577460

[B42] Utt A. Das P. K. Varjak M. Lulla V. Lulla A. Merits A. (2015). Mutations conferring a noncytotoxic phenotype on chikungunya virus replicons compromise enzymatic properties of nonstructural protein 2. *J. Virol.* 89 3145–3162. 10.1128/JVI.03213-14 25552719 PMC4337533

[B43] Utt A. Quirin T. Saul S. Hellstrom K. Ahola T. Merits A. (2016). Versatile trans-replication systems for Chikungunya virus allow functional analysis and tagging of every replicase protein. *PLoS One* 11:e0151616. 10.1371/journal.pone.0151616 26963103 PMC4786200

[B44] Utt A. Rausalu K. Jakobson M. Mannik A. Alphey L. Fragkoudis R. (2019). Design and use of Chikungunya virus replication templates utilizing mammalian and mosquito RNA polymerase I-mediated transcription. 93:e00794-19. *J. Virol.* 31217251 10.1128/JVI.00794-19 PMC6714806

[B45] van den Boom J. Meyer H. (2018). VCP/p97-mediated unfolding as a principle in protein homeostasis and signaling. *Mol. Cell.* 69 182–194. 10.1016/j.molcel.2017.10.028 29153394

[B46] van den Boom J. Wolf M. Weimann L. Schulze N. Li F. Kaschani F. (2016). VCP/p97 Extracts Sterically Trapped Ku70/80 Rings from DNA in Double-Strand Break Repair. *Mol. Cell.* 64 189–198. 10.1016/j.molcel.2016.08.037 27716483 PMC5161236

[B47] Varghese F. S. Rausalu K. Hakanen M. Saul S. Kummerer B. M. Susi P. (2017). Obatoclax inhibits alphavirus membrane fusion by neutralizing the acidic environment of endocytic compartments. *Antimicrob. Agents Chemother.* 61:e02227-e16. 10.1128/AAC.02227-16 27993855 PMC5328557

[B48] Varjak M. Saul S. Arike L. Lulla A. Peil L. Merits A. (2013). Magnetic fractionation and proteomic dissection of cellular organelles occupied by the late replication complexes of Semliki Forest virus. *J. Virol.* 87 10295–10312. 10.1128/JVI.01105-13 23864636 PMC3754020

[B49] Verma R. Oania R. S. Kolawa N. J. Deshaies R. J. (2013). Cdc48/p97 promotes degradation of aberrant nascent polypeptides bound to the ribosome. *eLife* 2:e00308. 10.7554/eLife.00308 23358411 PMC3552423

[B50] Wang T. Wang B. Huang H. Zhang C. Zhu Y. Pei B. (2017). Enterovirus 71 protease 2Apro and 3Cpro differentially inhibit the cellular endoplasmic reticulum-associated degradation (ERAD) pathway via distinct mechanisms, and enterovirus 71 hijacks ERAD component p97 to promote its replication. *PLoS Pathog.* 13:e1006674. 10.1371/journal.ppat.1006674 28985237 PMC5650186

[B51] Wolf D. H. Stolz A. (2012). The Cdc48 machine in endoplasmic reticulum associated protein degradation. *Biochim. Biophys. Acta.* 1823 117–124. 10.1016/j.bbamcr.2011.09.002 21945179

[B52] Wong H. H. Kumar P. Tay F. P. Moreau D. Liu D. X. Bard F. (2015). Genome-wide screen reveals valosin-containing protein requirement for coronavirus exit from endosomes. *J. Virol.* 89 11116–11128. 10.1128/JVI.01360-15 26311884 PMC4621105

[B53] Wu K. X. Phuektes P. Kumar P. Goh G. Y. Moreau D. Chow V. T. (2016). Human genome-wide RNAi screen reveals host factors required for enterovirus 71 replication. *Nat. Commun.* 7:13150. 10.1038/ncomms13150 27748395 PMC5071646

[B54] Ye Y. Meyer H. H. Rapoport T. A. (2003). Function of the p97-Ufd1-Npl4 complex in retrotranslocation from the ER to the cytosol: dual recognition of nonubiquitinated polypeptide segments and polyubiquitin chains. *J. Cell Biol.* 162 71–84. 10.1083/jcb.200302169 12847084 PMC2172719

[B55] Ye Y. Tang W. K. Zhang T. Xia D. (2017). A mighty “protein extractor” of the cell: structure and function of the p97/CDC48 ATPase. *Front. Mol. Biosci.* 4:39. 10.3389/fmolb.2017.00039 28660197 PMC5468458

[B56] Yi Z. Fang C. Zou J. Xu J. Song W. Du X. (2016). Affinity purification of the hepatitis C virus replicase identifies valosin-containing protein, a member of the ATPases associated with diverse cellular activities family, as an active virus replication modulator. *J. Virol.* 90 9953–9966. 10.1128/JVI.01140-16 27558430 PMC5068543

[B57] Yi Z. Yuan Z. (2017). Aggregation of a hepatitis C virus replicase module induced by ablation of p97/VCP. *J. Gen Virol.* 98 1667–1678. 10.1099/jgv.0.000828 28691899

[B58] Zeiler M. Straube W. L. Lundberg E. Uhlen M. Mann M. (2012). A protein epitope signature tag (PrEST) library allows SILAC-based absolute quantification and multiplexed determination of protein copy numbers in cell lines. *Mol. Cell. Proteomics* 11:O111009613. 10.1074/mcp.O111.009613 21964433 PMC3316735

[B59] Zhang J. Lan Y. Sanyal S. (2017). Modulation of lipid droplet metabolism-a potential target for therapeutic intervention in flaviviridae infections. *Front. Microbiol.* 8:2286. 10.3389/fmicb.2017.02286 29234310 PMC5712332

[B60] Zhang X. Wang Y. (2015). Cell cycle regulation of VCIP135 deubiquitinase activity and function in p97/p47-mediated golgi reassembly. *Mol. Biol. Cell* 26 2242–2251. 10.1091/mbc.E15-01-0041 25904330 PMC4462942

