# Supplementary figures and images for: VCP/p97 Is a Proviral Host Factor for Replication of Chikungunya Virus and Other Alphaviruses
Source: Front Microbiol. 2019 Sep 24;10:2236. doi: 10.3389/fmicb.2019.02236 (PMC6787436; doi:10.3389/fmicb.2019.02236)

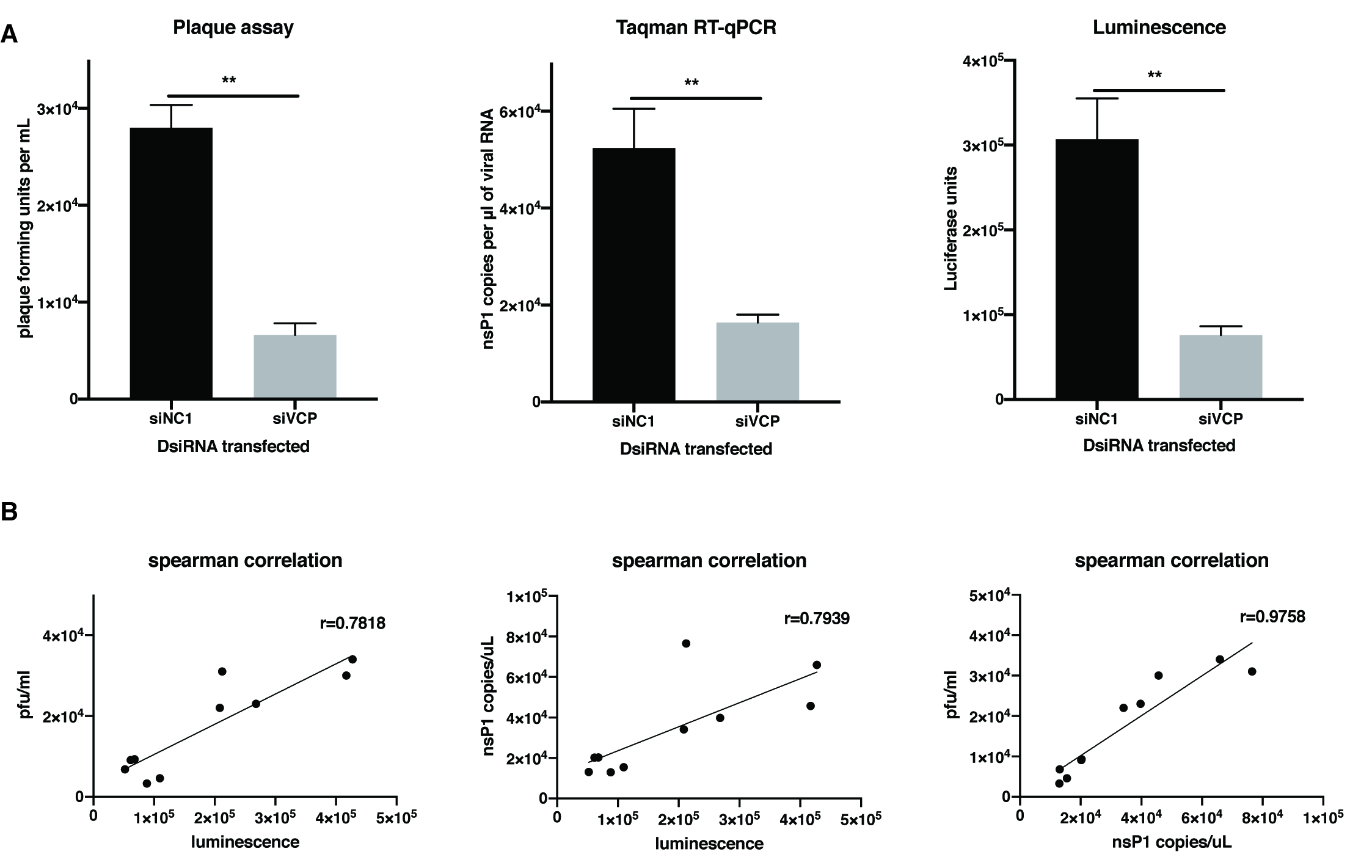

Supplement: FIGURE S1 — Viral RNA quantification, plaque assay, and luciferase luminescence show similar results. A hundred thousand HEK 293T cells per well were transfected with siRNA at 10 nM for 48 h. Cells were then infected with 10,000 pfu of Gluc-tagged CHIKV-LR. (A) At 16 hpi, supernatant was collected from each well for viral RNA RT-qPCR, plaque assay and luminescence reading. (B) Spearman correlations of the different readings for the results presented in panel A. Results are from two independent experiment with 2–3 well per condition in each experiment. [file Image_1.TIF]

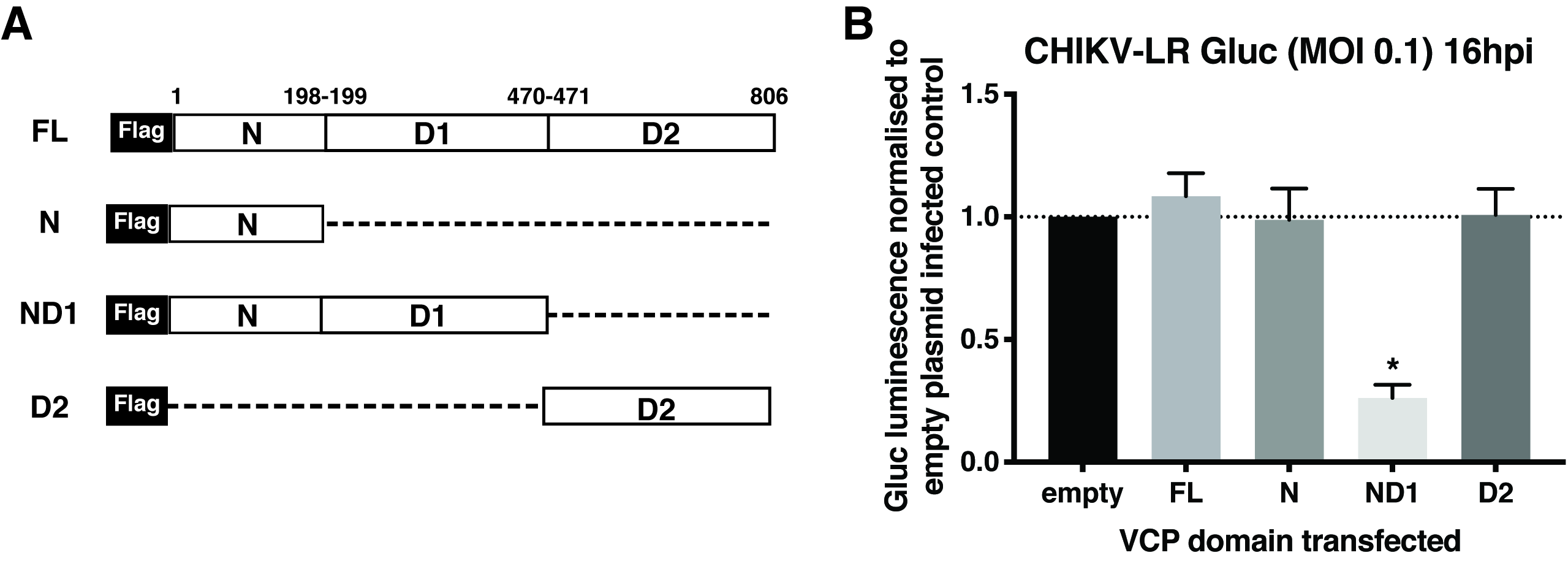

Supplement: FIGURE S2 — (A) The schematic representation of flag-tagged VCP and VCP domain constructs. (B) HEK293T were plated in 96-well format at 30,000 cells per well. At 1 day post-plating, cells were transfected with 100 ng per well of indicated VCP constructs or empty plasmid control. At 1 day post-transfection, cells were infected using 3,000 pfu per well of Gluc-tagged CHIKV and incubated for 16 h. At which time, 50 μL of supernatant was collected for luciferase assay. Data of panel B are representative of four independent experiment presented as mean ± SD and analyzed by Mann–Whitney non-parametric two-tailed test; ∗p < 0.05. [file Image_2.TIF]
